# Hashimoto's Thyroiditis: Similar and Dissimilar Characteristics in Neighboring Areas. Possible Implications for the Epidemiology of Thyroid Cancer

**DOI:** 10.1371/journal.pone.0055450

**Published:** 2013-03-18

**Authors:** Adele Latina, Damiano Gullo, Francesco Trimarchi, Salvatore Benvenga

**Affiliations:** 1 Endocrinology Division, Department of Clinical and Molecular Biomedicine, University of Catania Medical School, Garibaldi-Nesima Hospital, Catania, Italy; 2 Section of Endocrinology, Experimental Clinical Department of Medicine and Pharmacology, University of Messina, Messina, Italy; 3 Master Program of Childhood, Adolescent, and Women's Endocrine Health, University of Messina, Messina, Italy; University of Bari, Italy

## Abstract

**Context:**

Medical centers worldwide report an increased frequency of Hashimoto's thyroiditis (HT) and thyroid cancer (TC), two environmentally influenced diseases. In Sicily, data on HT are available for the province of Messina (1975–2005); data on TC are available for the whole island (2002–2004), with the volcanic province of Catania having the highest incidence.

**Objective:**

To replicate in Catania, on comparable years, the HT data of Messina.

**Design, Methods, Setting:**

Review of the clinical records of patients in years 1995–2005 to compare presentation and yearly changes of HT. During 1995–2005, records were computer stored in the Endocrine Divisions of the University Hospitals of Catania and Messina, two tertiary referral centers.

**Results:**

Catania is outnumbered by Messina (742 *vs.* 3,409 HT patients). Similar were the linear increase in the yearly number of HT patients, rates of thyroid dysfunctions though with different proportions of subclinical and overt hypothyroidism, and rates of positiveness for TgAb or TPOAb. Different were age and its yearly trend; gender distribution and rates of the sonography variants, though yearly trends were similar.

**Conclusion:**

The HT epidemics is smaller in Catania, with changes in presentation overlapping partially those in Messina. Whatever environmental factors might be involved, they (and/or their intensity) were not necessarily the same in these provinces. Intriguingly, the expected number of TC in HT patients with thyroid nodules in Catania is congruent with that of the general population of this province, but it is far less than in the Messina province. Thus, TC and HT incidences could be influenced by distinct environmental factors.

## Introduction

Of the endocrine glands, the thyroid is the leader in the autoimmune and neoplastic domains, as Hashimoto's thyroiditis (HT) and thyroid cancer (TC) are the leading endocrine autoimmune disease and endocrine malignancy [1]–[4]. HT may coexist with TC, particularly the papillary histotype (PTC), but its link is still controversial (predisposing?, protective?, neutral?) [2]–[5], though there is some agreement on the more favorable prognosis of PTC when it is associated with HT. Both diseases are under the predisposing/promoting influence of exogenous factors, and it is to environmental changes that can be attributed the increased frequency and/or geographically different epidemiology of either disease noticed at several medical centers [1]–[11]. It is well known that HT is a leading cause of acquired hypothyroidism even in childhood [9] and its frequency increases with age, so that occult HT and related initial impairment of thyroid function is responsible for the upward trend of the TSH upper limit as age advances [10]. Based on the large NHANES III survey, serum thyroid antibodies (TPOAb and/or TgAb) were detected in 12.5% of the population (for refs. see 6). When both TPOAb and TgAb were present, the odds ratio for overt hypothyroidism was 23.5 and for subclinical hypothyroidism was 11.7. These were higher than for TPOAb alone for overt hypothyrodism (OR = 6.9) and for subclinical hypothyroidism (OR = 4.0).

Recently, Benvenga and Trimarchi [8] reported that the frequency of HT at the Division of Endocrinology at the University Hospital of Messina (northeastern Sicily) increased enormously over the years 1975–2005. Indeed, there were ≈33 new HT patients per year in the 5-year period 1975–79 as compared with ≈444 in the 5-year period 2001–05. The increased local incidence of HT was confirmed on other patients, namely patients referred to the Cytology Unit of the same hospital for the fine-needle aspiration cytology (FNAC) of a single or dominant thyroid nodule over the period 1988–2007 [9]. Cytological diagnoses of HT were one in the period 1988–92, but 56 in 2003–05. Importantly, there was no increased frequency in the cytological diagnosis of the non-autoimmune inflammations, that is, de Quervain's and Riedel's thyroiditis [9]. This increased number of HT patients was accompanied by changes in other parameters, leading to the conclusion that environmental modifications might have accounted for the changed presentation of HT [6].

On the other hand, the Division of Endocrinology at the University Hospital of Catania (the main city of the homonymous province just south of the Messina province) led a region-based epidemiologic survey of TC in Sicily that covered the period 2002–2004 [11]. This study found that TC, 90% of which being PTC, had an incidence greater than previously thought and with a remarkable geographical peculiarity, in that the Catania province had the greatest incidence of TC [11]. These data were related to environmental issues, because the Catania province contains the active Etna volcano within its borders.

Keeping in mind the controversial link between HT and TC association, and having the daily practice given the hint that the local frequency of HT had increased at the Division of Endocrinology in Catania, we sought to replicate the Messina study [6]. In other terms, we wished to test whether the Catania cohort of HT was similar to or dissimilar from the Messina cohort.

## Patients and Methods

### Patients

Similar to the previous work [6], this is a retrospective study. Because clinical records at the Thyroid Clinic of the Endocrine Division in Catania were computer stored starting in January 1995, we reviewed the clinical records of the approximately 49,000 thyroid patients who were admitted from January 1, 1995 through December 31, 2009. To compare reliably the two cohorts, the methodology was the same as that described detailedly for Messina [6]. Thus, we first reviewed the individual diagnosis to select the total number of 1,453 HT patients admitted from January 1995 through December 2009. However, to permit a homogeneous comparison with the Messina cohort, results emphasized the overlapping period of the years 1995–2005, when a total of approximately 28,000 patients with thyroid problems were observed in Catania, as compared to approximately 11,000 patients in Messina. More precisely, we assessed the yearly changes of these indices: number of new HT patients; age at presentation; F∶M ratio; thyroid size and nodule(s); functional status (euthyroidism, hypothyroidism, transient hyperthyroidism, Hashitoxicosis); and TgAb and TPOAb status.

**Figure 1:**
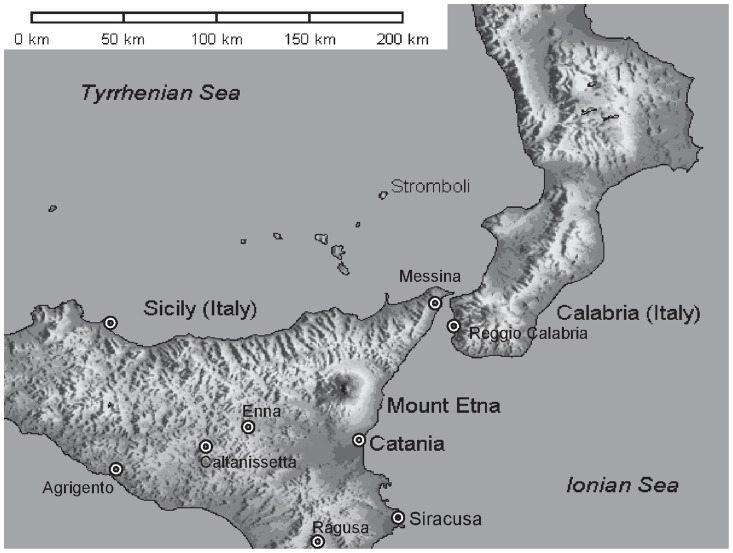
Geographic location of the Catania and Messina provinces. Approximately, 14% of the Catania cohort is represented by HT patients coming from the neighboring provinces of Siracusa (6%), Ragusa (2.5%), Enna (2%), Caltanissetta (2.5%) and Agrigento (0.7%). This compares with approximately 18% of the Messina cohort of HT patients coming from Southern Calabria, particularly from the province of Reggio Calabria. Population in these provinces is approximately 1.1 million (Catania), 660,000 (Messina), 400,000 (Siracusa), 300,000 (Ragusa), 275,000 (Caltanissetta), 175,000 (Enna), 450,000 (Agrigento), and 560,000 (Reggio Calabria).

The geographic position of the two provinces and the percent contribution from neighboring provinces to the study groups are illustrated in the Figure 1.

### Diagnosis of HT

As reported earlier [6], HT was diagnosed based on a combination of clinical, ultrasonography and biochemical data. Clinical data consisted of palpation of a firm, rubbery, and fine granular thyroid. Sonography was performed to detect the typical HT pattern of heterogeneous structure with diffuse or patchy hypoechogenicity, to measure thyroid size and to probe the presence of thyroid nodules. The sonographic variants (or categories), based on thyroid size and nodularity disregarding pseudonodules were: (i) atrophic (thyroid of reduced size); (ii) nongoitrous/nonnodular (thyroid of normal size with no nodules); (iii) nongoitrous/nodular (thyroid of normal size, but with one or more nodules); (iv) goitrous/nonnodular (thyroid of enlarged size with no nodules), (v) goitrous/nodular (thyroid of enlarged size and with one or more nodules). To maximize the number of variants, two additional categories were considered: the “goitrous, regardless of nodules”, which results from pooling the fourth and fifth variant, and the “nodular, regardless of goiter”, which results from pooling the third and the fifth variant.

Biochemical data consisted of evidence for euthyroidism, hypothyroidism (either subclinical or overt) or self-limiting hyperthyroidism [6]. Subclinical (or mild, initial) hypothyroidism was defined by increased TSH coexisting with normal free T4 (FT4); overt hypothyroidism was defined by increased TSH coexisting with subnormal T4 or FT4. Euthyroidism did not exclude HT, if there were other indicators (TgAb and/or TPOAb positiveness and abnormal sonography).

### Ethics statement

Data were retrieved from database records of patients evaluated at the Thyroid Clinic of the Division of Endocrinology of the Garibaldi-Nesima Hospital, Catania. The Local Ethics Committee approved the study and did not require the patient's informed consent as data were analyzed anonymously.

### Methods

Assays of serum FT3, FT4, TSH, TgAb and TPOAb for the Messina cohort were described previously [6]. Particularly, the kits for thyroid antibodies were the immunoradiometric assays by DiaSorin (Saluggia, Italy), with normal values <100 (TgAb) or <10 U/ml (TPOAb). For the Catania cohort, assays were disparate since it is not the policy of that endocrine division to repeat consistent results that are exhibited by patients.

### Statistics

Data are mean±SD. We compared continuous variables using Student t test, and proportions of categorical variables with the chi-square test (χ^2^). When variables had nongaussian distribution, values were log-10 transformed prior to analysis. To test for trend of changes of a given index over the years, we calculated a correlation coefficient between the index and calendar years by linear correlation. Data were analyzed with the Prism software package (GraphPad, San Diego, USA).

## Results

**Figure 2:**
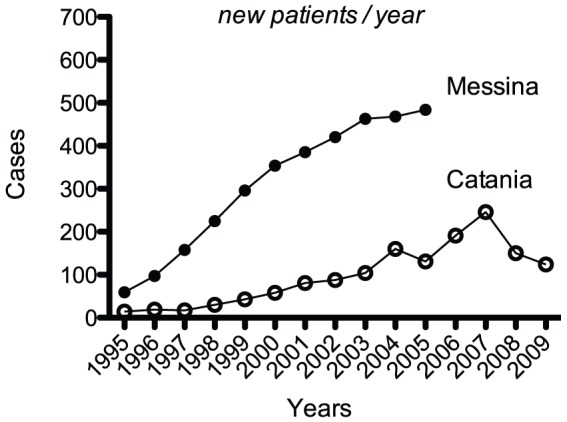
Number of the Hashimoto's thyroiditis patients in the cohorts from Catania and Messina. In this and subsequent figures, data from the Messina cohort are from ref. 6.

**Figure 3:**
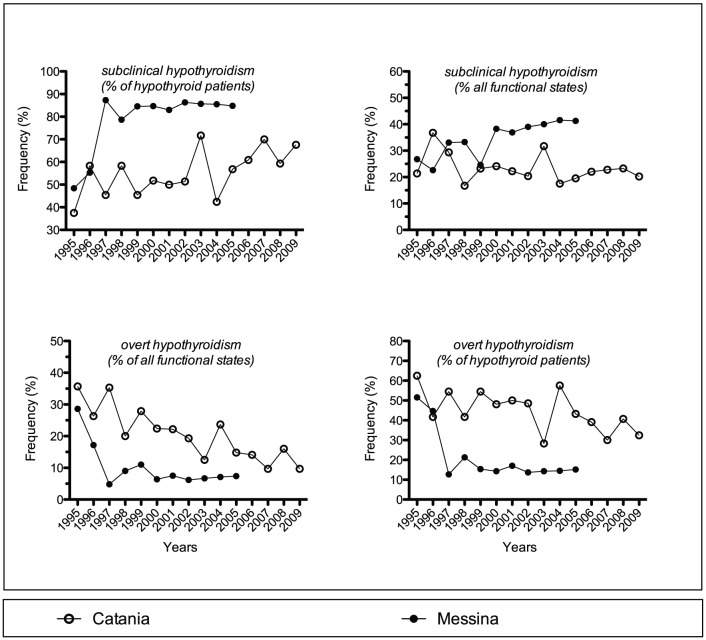
Yearly prevalence of the indicated functional states in the two Hashimoto's thyroiditis cohorts of patients.

**Figure 4:**
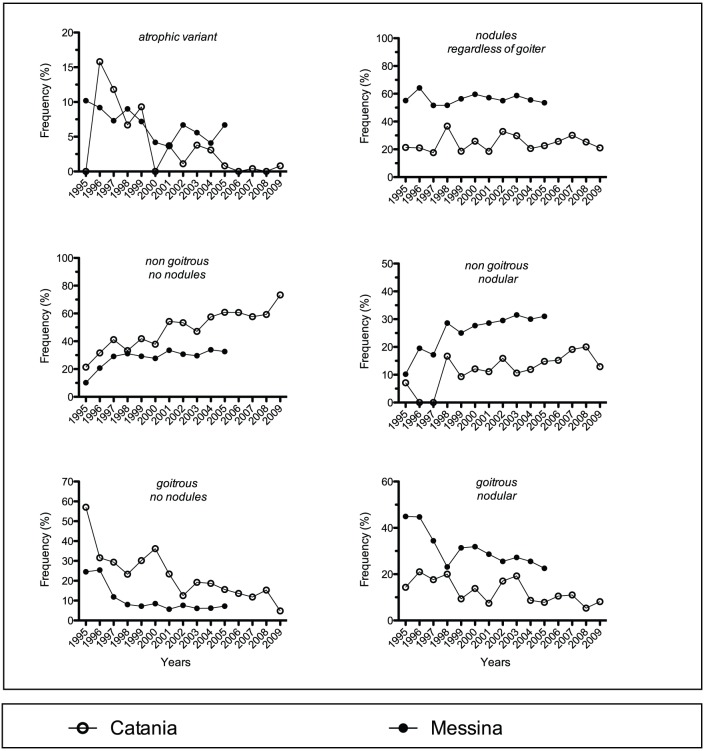
Yearly prevalence of the indicated variants of Hashimoto's thyroiditis (HT) based on thyroid size and nodules as assessed by thyroid ultrasonography in the two HT patients cohorts.

**Figure 5:**
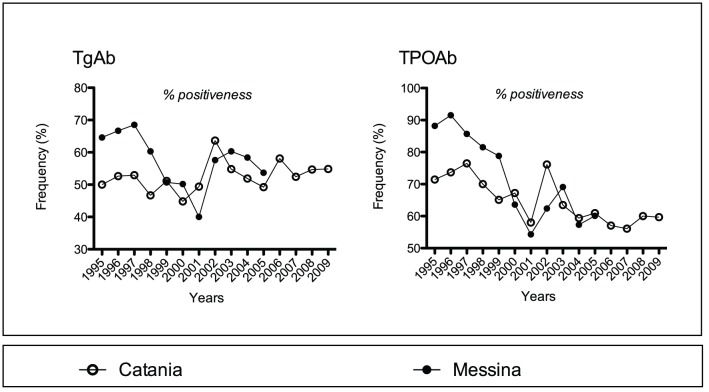
Yearly prevalence of the serum positiveness for thyroglobulin autoantibodies (TgAb, left panel) and thyroperoxidase autoantibodies (TPOAb, right panel), *viz.* proportion of patients with higher-than-normal serum levels of TgAb and TPOAb, in the two cohorts of patients with Hashimoto's thyroiditis.

Data are summarized in Tables 1 and illustrated in Figure 2, Figure 3, Figure 4, and Figure 5. Figures emphasize results that were statistically different in the two cohorts.

**Table 1:** Clinical, biochemical and ultrasonographic characteristics of Hashimoto's thyroiditis patients observed at two endocrine divisions (University Hospital of Catania or Messina) during the indicated years.

Characteristics	Catania		Messina	Statistics
	1995–2009	1995–2005	1995–2005	1995–2005
	(n = 1453)	(n = 742)	(n = 3409)	Catania *vs.* Messina
New patients per year (n)	97.87±69.8	67.45±49.0^a^	309.90±154.0^a^	P = **0.0036** a
Gender, % of females and % of males	90.9 and 9.1%	92.6 and 7.4%	89.5 and 10.5%	χ^2^ = 6.600, P = **0.010**
Gender, Female to male ratio (n∶1)	11.7	12.5	8.5	P = 0.287^a^
	12.69±6.93	14.09±7.44^a^	11.15±4.9^a^	
Age in years at presentation (mean)	42.2±14.7	42.3±14.5^a^	41.6±2.4^a^	P = **0.01** a
[median]	[42]	[42]	[41]	
Hyperthyroid. (prevalence, % of all pts)	2.5	2.6^b^	2.1^b^	χ^2^ = 0.747, P = 0.387^b^
	1.98±2.39	1.89±2.80^a^	2.54±1.79^a^	P = 0.361^a^
Euthyroidism (prevalence, % of all pts)	50.2	54.6^b^	52.2^b^	χ^2^ = 1.218, P = 0.270^b^
	53.69±9.95	50.20±9.12^a^	52.0±5.89^a^	P = 0.583^a^
Hypothyroidism (prevalence, % of all patients)	38.7	42.8^b^	45.7^b^	χ^2^ = 0.51, P = 0.476^b^
	44.05±10.73	47.55±10.28^a^	45.42±4.72^a^	P = 0.690^a^
Subclinical hypothyroidism	58.7	52.8^b^	82.6^b^	χ^2^ = 112.4, P = **2.9×10^−26^** b
(prevalence, % of hypo patients)	55.12±10.14	51.73±9.40^a^	78.66±13.53^a^	P = **2.6×10^−5^** a
Overt hypothyroidism	48.2	47.2^b^	17.4^b^	χ^2^ = 112.4, P = **2.9×10^−26^** b
(prevalence, % of hypo patients	44.86±10.12	48.25±9.39^a^	21.33±13.53^a^	P = **2.6×10^−5^** a
Atrophic variant (prevalence, %)	1.8	3.4^b^	5.9^b^	χ^2^ = 7.71, P = **0.0055** b
	3.82±4.94	5.10±5.23^a^	6.71±2.20^a^	P = 0.328^a^
Nongoitrous, nonnodular variant (prevalence, %)	48.8	50.7^b^	30.5^b^	χ^2^ = 110.6, P = **7.2×10^−26^** b
	48.77±13.95	43.68±12.24^a^	28.03±6.92^a^	P = **0.0014** a
Nongoitrous, nodular variant (prevalence, %)	14.5	12.0^b^	27.9^b^	χ^2^ = 81.8, P = **1.5×10^−19^** b
	11.78±5.93	9.95±5.67^a^	24.36±8.20^a^	P = **0.0001**
Goitrous, nonnodular variant (prevalence, %)	16.8	21.5^b^	7.7^b^	χ^2^ = 127.7, P = **1.3×10^−29^** b
	22.85±12.83	27.02±12.35	9.89±5.69	P = **0.00046** a
Goitrous, nodular variant (prevalence, %)	10.8	12.4^b^	28.0^b^	χ^2^ = 78.8, P = **6.9×10^−19^** b
	12.73±5.17	14.19±5.16^a^	30.88±7.80^a^	P = **8.6×10^−6^** a
Goitrous, regardless of nodules (prevalence, %)	27.6	33.9^b^	35.7^b^	χ^2^ = 0.83, P = 0.361^b^
	35.58±15.36	41.21±13.86^a^	40.77±13.05^a^	P = 0.940^a^
Nodular, regardless of goiter (prevalence, %)	25.3	24.4^b^	55.9^b^	χ^2^ = 241.7, P = **3.2×10^−54^** b
	24.51±5.70	24.15±6.38^a^	54.84±3.62^a^	P = **4×10^−8^** a
TgAb (prevalence of +ve cases, %)	53.5	52.2^b^	55.1^b^	χ^2^ = 2.11, P = 0.146^b^
	52.48±4.58	51.56±4.92^a^	57.36±8.23^a^	P = ***0.060*** a
TPOAb (prevalence of +ve cases, %)	61.2	64.4^b^	66.6^b^	χ^2^ = 1.28, P = 0.258^b^
	64.98±7.06	67.44±6.60^a^	72.04±13.05^a^	P = 0.551^a^
TgAb (levels in U/ml, mean±SD)	1155±3708	1101±2472	922±1821	N/A^c^
[median]	[370]	[389]	[237]	
TPOAb (levels in U/ml, mean±SD)	1455±2980	1692±3092	625±1211	N/A^c^
[median]	[498]	[578]	[174]	

In 2005, the population was 1,071,883 in the province of Catania and 657,785 in the province of Messina (http://demo.istat.it/pop2009/index.html). During the period 1995–2005, the total number of thyroid patients referred to the two endocrine divisions approximated 28,000 (Catania) and 11,000 (Messina). In the period 1995–2009 the total numbers of thyroid patients referred to the endocrine division in Catania approximated 49,000. Though our study was not (and was not intended to be) a province-wide screening for HT, in order to give an approximation of the province population-adjusted number of the thyroid patients and HT patients in Catania vs. Messina assuming that all such patients living in the two provinces were referred to the two institutions, the total number of thyroid patients was 2,612 vs. 1,672 per 100,000 (a 1.6-fold difference) while the number of HT patients was 69 vs. 518 per 100,000 (a 7.5-fold difference).

Data are reported as weighted averages, mean ± SD [and median]. P values<0.05 are typed boldface. P values between 0.05 and 0.10 are typed boldface italics.

a Comparison between means was made by the Student t-test.

b Comparison between rates (weighted averages) was examined by the chi square (χ^2^) test.

c Comparison between serum levels of TgAb or TPOAb missing because of differences in assays (see text, Patients and Methods).

### Epidemiology

The Catania cohort consists of 1,453 HT patients of whom 742 observed in 1995–2005 (Table 1). This number is 4.6-fold less than the 3,409 HT patients of Messina during 1995–2005, but 12-fold less in percentage terms (2.6% *vs.* 31%), as the total number of thyroid patients observed in 1995–2005 approximated 28,000 and 11,000, respectively. In both the Catania and Messina cohorts the number of HT patients increased at a similar highly significantly linear rate over the period 1995–2005 (r = 0.953, P = 5.7×10^−6^, and r = 0.997, P = 1.5×10^−7^) (Table 2; Figure 2). However, the increase started in the year 1998 in Catania, but at least 3 years earlier in Messina (see Figure 1 in ref. 6).

**Table 2:** Summary of the relationships between the calendar year (1995–2005) and the indicated characteristic in the Catania or the Messina cohort of Hashimoto's thyroiditis (HT) patients.

Characteristics	Catania series (1995–2005)		Messina series (1995–2005)	
	r (95% C.I.)	P	r (95% C.I.)	P
New patients per year (n)	0.953 (0.824 to 0.988)	**5.7×10^−6^**	0.979 (0.920 to 0.995)	**1.5×10^−7^**
Female to male ratio (n/1)	0.224 (−0.434 to 0.726)	0.508	−0.865 (−0.964 to −0.551)	**0.00006**
Age at presentation (mean)	0.575 (−0.435 to 0.726)	***0.064***	0.183 (−0.467 to 0.706)	0.183
Age at presentation (median)	0.088 (−0.541 to 0.653)	0.797	0.420 (−0.240 to 0.814)	0.199
Hyperthyroidism (prevalence, % of all pts.)	0.472 (−0.178 to 0.836)	0.142	−0.497 (−0.844 to 0.146)	0.12
Euthyroidism (prevalence, % of all pts.)	0.744 (0.261 to 0.929)	**0.0086**	−0.019 (−0.612 to 0.587)	0.955
Hypothyroidism (prevalence, % of all pts.)	−0.817 (−0.951 to −0.425)	**0.0021**	0.215 (−0.442 to 0.722)	0.526
Subclinical hypo (prevalence % of hypo pts)	0.329 (−0.338 to 0.776)	0.323	0.694 (0.161 to 0.913)	**0.0178**
Overt hypo (prevalence, % of hypo pts)	−0.329 (−0.776 to 0.338)	0.324	−0.694 (−0.913 to −0.161)	**0.0178**
Atrophic variant (prevalence,%)	−0.505 (−0.848 to 0.137)	0.113	−0.703 (−0.916 to −0.178)	**0.0158**
Nongoitrous/nonnodular variant (preval., %)	0.915 (0.697 to 0.978)	**<0.0001**	0.740 (0.252 to 0.928)	**0.0092**
Nongoitrous/nodular variant (prevalence, %)	0.628 (0.0454 to 0.892)	**0.0384**	0.859 (0.533 to 0.963)	**0.0007**
Goitrous/nonnodular variant (prevalence, %)	−0.777 (−0.939 to −0.331)	**0.0049**	−0.764 (−0.935 to −0.303)	**0.0062**
Goitrous/nodular variant (prevalence, %)	−0.496 (−0.844 to 0.148)	0.121	−0.805 (−0.947 to −0.398)	**0.0028**
Goitrous, regardless of nodules (preval., %)	−0.876 (−0.967 to 0.584)	**0.0004**	−0.816 (−0.905 to −0.425)	**0.0022**
Nodular, regardless of goiter (prevalence, %)	0.157 (−0.488 to 0.619)	0.644	0.348 (−0.318 to 0.784)	0.294
TgAb (prevalence of +ve cases,%)	0.190 (−0.463 to 0.709)	0.576	−0.473 (−0.836 to 0.177)	0.142
TPOAb (prevalence of +ve cases, %)	−0.656 (−0.901 to −0.092)	**0.028**	−0.874 (−0.967 to −0.576)	**0.00044**
TgAb (levels in U/ml, mean)	0.447 (−0.209 to 0.825)	0.168	−0.804 (−0.947 to −0.395)	**0.0028**
TgAb (U/ml, log-10 transf, mean)	0.401 (−0.262 to 0.807)	0.221	−0.880 (−0.969 to −0.594)	**0.00035**
TgAb (levels in U/ml, median)	−0.356 (−0.788 to −0.310)	0.282	−0.683 (−0.910 to −0.140)	**0.020**
TPOAb (levels in U/ml, mean)	0.325 (−0.341 to 0.774)	0.329	−0.821 (−0.952 to −0.437)	**0.0019**
TPOAb (U/ml, log-10 transf, mean)	0.511 (−0.128 to 0.850)	0.108	−0.906 (−0.976 to −0.671)	**0.00012**
TPOAb (levels U/ml, median)	0.083 (−0.544 to 0.651)	0.808	−0.689 (−0.912 to −0.152)	**0.019**

P values<0.05 are typed boldface.

### Gender and age

In the period 1995–2009, the Catania cohort consisted of 1,320 females and 133 males (F∶M ratio = 9.9∶1). However, in 1995–2005, the Catania cohort consisted of 687 females and 55 males (F∶M ratio = 12.5∶1), as compared with 3,050 females and 359 males (F∶M ratio of 8.5∶1) in the Messina cohort. As a result, the percentage distribution of the two genders differed in the two cohorts (χ^2^ = 6.600, P = 0.010) (Table 1). The HT women in Messina outnumbered the HT women in Catania by 4.4 times, while HT men did so by 6.5 times. The gap narrowed to 3.7 (women) and 5.9 times (men) in the period 2002–2004, when there were 327 females and 26 males in Catania (F∶M ratio = 12.6∶1) and 1,198 females and 153 males in Messina (F∶M ratio = 7.8∶1). These three years are of interest for the considerations on the different incidence of TC in Sicily (see Discussion). If years 1995–1997 are omitted from the period 1995–2009 due to fewer than 20 HT patients/year in the Catania cohort, the yearly trend of the F∶M ratio becomes significant (r = −0.711, P = 0.0096, instead of r = 0.224, P = 0.508 for the period 1995–2005), and it becomes close to the corresponding Messina's correlation (r = −0.865, P = 0.00059) (Table 2). The Catania F∶M ratio of 7.0∶1 for years 2007–09 matched the Messina 7.3∶1 ratio for years 2003–05 [6].

Both the mean and median age were in the early 40's, the Catania cohort being one year older than the Messina cohort (P = 0.01) (Table 1).

### Functional status

Distribution of HT patients between euthyroidism, hypothyroidism and hyperthyroidism did not differ in Catania (54.4%, 43.0% and 2.6%) *vs.* Messina (52.2%, 45.7% and 2.1%) (Table 1). However, rates of subclinical and overt hypothyroidism within the hypothyroidism category differed (56.6 and 43.4% in Catania *vs.* 82.6 and 17.4% in Messina, χ^2^ = 112.4, P = 2.9×10^−26^). As a result, the annual prevalence of overall hypothyroidism trended upward insignificantly in Messina, while it decreased significantly in Catania (Table 2 and Figure 3).

### Thyroid size and nodules (ultrasonographic variants)

The two cohorts differed in rates for all variants except “goiter, regardless of nodules” (Table 1). The rate of the variant “nodularity, regardless of goiter” in Catania was 2.3-fold lower than in Messina (Table 1). Excluding these two variants, cohorts agreed in nongoiter/nonnodularily ranking first (with great inter-cohort difference) and atrophy ranking last (with little inter-cohort difference), but the hierarchy and rates of the other variants differed much (Table 1). In Catania, the rank was nongoiter/nonnodularity, goiter/nonnodularity, goiter/nodularity, nongoiter/nodularity and atrophy with a ratio of 14.9∶6.3∶3.6∶3.5∶1. In Messina, the rank was nongoiter/nonnodularity, goiter/nodularity, nongoiter/nodularity, goiter/nonnodularity and atrophy with a ratio of 5.2∶1.3∶4.7∶4.7∶1. Each variant displayed the same upward or downward trend over time in the two cohorts (Table 2; Figure 4). The variant “nodular, regardless of goiter” increased insignificantly, and steeperly in Messina (r = 0.348 *vs.* r = 0.157). Otherwise, the trend was statistically significant in either cohort, except for the atrophic and goitrous/nodular variants in Catania, as they only approached borderline significance (P = 0.113 and 0.121, respectively). The variants with very similar trends in the Catania *vs.* the Messina cohort were the goitrous/nodular and the “goitrous, regardless of nodules” (r = −0.777 *vs.* −0.764, and r = −0. 876 *vs.* −0.816) (Table 2).

### Serum thyroid autoantibodies (TgAb, TPOAb)

Prevalence of positiveness for TgAb or TPOAb was greater in Messina until 1998 or 1999 (Figure 5). Thereafter, prevalences were very similar in the two cohorts. This similarity, which was striking for TPOAb, is also supported by the cohorts sharing the transient peak following the nadir in 2001. The positiveness rate during 1995–2005 trended downward for TPOAb in either cohort, but less steeperly in Catania (r = −0.656, P = 0.028 *vs.* r = −0.874, P = 0.00044), and it went in opposite directions for TgAb (r = 0.190, P = 0.576 vs. r = −0.473, P = 0.142) (Table 2). Though serum levels are incomparable due to differences in kits, they fluctuated in Catania but fell in Messina. As a result, the yearly trend of median levels was downward, and statistically significant only in Messina.

## Discussion

Here we have shown that there are similarities and dissimilarities between HT patients from neighboring provinces, and that in Catania HT indices fluctuated more over time. Importantly, dissimilarities cannot be explained by the mere 1-year difference of age. Moreover, in either cohort the correlation between age and calendar year was direct (steeper in Catania), not inverse.

Messina outnumbers Catania markedly, though the magnitude of the linear increase in the number of HT patients during 1995–2005 was similar. Independent data from the Messina's Cytology Unit [9], confirm the striking difference. In 1995–2005 there were 334 persons, who were referred to that Unit for the FNAC of a single or dominant nodule, were cytologically diagnosed as HT. Obviously, these 334 patients had the nodular variant of HT. Though the cytological [7] and the endocrine [6] Messina cohorts contain distinct HT patients, one can estimate that ≈18% of HT patients (334/1,894) have thyroid nodules worthy of FNAC. By comparison, during the same years (1995–2005), in the Catania endocrine cohort there were 181 HT patients with “thyroid nodules, regardless of goiter”. This is 10-fold less than the 1,894 equivalent patients in the Messina endocrine cohort. If the 18% rate is applied to the 181 HT patients, then the 10-fold difference seen in the endocrine setting holds true in a cytological setting (334 in Messina vs. 33 in Catania; 0.18×181 = 33). As the frequency of thyroid nodules increases with age, it is noteworthy that the greater rate of this variant in Messina cannot be explained by an older age of patients in this variant, since both median and mean age matched (46 and 46.4 *vs.* 46 and 46.4 years in Catania).

Reference to this FNAC cohort [7] underscores the consistently lower F∶M ratio of Messina (8.0∶1 in 1995–2005, confirming the 8.5∶1 in the 1995–2005 endocrine cohort) compared to Catania (15.5∶1, in the 1995–2005 variant “thyroid nodules, regardless of goiter”). To stress further the gender issue, the difference between the endocrine cohorts was amplified by the opposite direction of the trend of the F∶M ratio over the years (Table 2).

For some similarities Catania lagged behind Messina. For instance, the increase in yearly frequency of HT was appreciable in 1998 (+76% over year 1997), but in Messina it was appreciable earlier (+100% in 1992 over 1991 and +64% in 1996 over 1995) [6]. There were three consecutive years with a high F∶M ratio (≈20∶1) preceded by two consecutive years with a very low F∶M ratio (≈4∶1 to 6∶1). The three years were 1997–99 in Catania, and 1995–97 in Messina [6], while the two years were 1995–96 and 1993–94 [6]. The mean F∶M ratio of 7.0∶1 for years 2007–09 in Catania matches the 7.3∶1 ratio for years 2003–05 in Messina. Finally, there were two peaks of hyperthyroidism: years 2000 and 2005 in Catania (not shown), but 1991 and 1995 in Messina [6]. Perhaps, these delays indicate retarded exposure to the same environmental factor(s). It is of interest that the two peaks of hyperthyroidism coincided or almost coincided with a rebound in the positivity rate of both TgAb and TPOAb and an increased mean level of TgAb and/or TPOAb (Figure 5 of this manuscript, and Figure 4 and 5 of ref. 6). Due to the retrospective nature of our study, we have no clues on the possible environmental factors that were responsible for such two peaks. However, the parallel changes in serum levels of thyroid autoantibody levels suggest that the chemicals (or mixture of chemicals) involved had to be internalized by the thyroid, causing a transient leakage of colloid-stored thyroid hormones and increasing the immunogenicity of thyroglobulin and thyroperoxidase. These chemicals could be released in the environment periodically or, if released continuously, their concentration in the environment can increase periodically.

Because of the autoimmune nature of HT, it is of interest to compare serum TgAb and TPOAb in the two cohorts. From the parallel changes shown in Figure 5, including the striking chronological coincidence of peaks or nadir values, one has to conclude that the two cohorts shared environmental factors that prompted those changes. The difference between the two cohorts lies, probably, in the intensity of exposure to these external factors. A stronger intensity of exposure would be more functionally destructive for the follicular epithelium and thus cause a greater proportion of overt thyroid failure (as observed in the Catania HT series) compared to a lighter intensity of exposure that would cause a milder functional impairment or subclinical thyroid failure (as observed in the Messina HT series). The downward trend of TgAb and TPOAb in terms of both positiveness and serum concentration, all trends being more marked in the Messina cohort, would suggest that the shared environmental factors might have chemically modified the major autoantigenic epitopes of both Tg and TPO and rendered more exposed minor and hidden epitopes, such as the hormonogenic domains of Tg. This hypothesis was put forward by the Messina endocrinologists to explain why the downward trend of TgAb positiveness over time coincided with an upward trend of positiveness for thyroid hormone antibodies [6], [12].

During the years 2002–06, the epidemiology of TC has not changed in Sicily, including the comparison between the Catania and Messina provinces [11]. In contrast, during the same period the number of HT patients in Catania doubled (from 88 to 191), and a number of rates increased/decreased by ≈10 percent points: mild or overt hypothyroidism within hypothyroid patients (51 to 61% or 49 to 39%), nongoiter/nonnodularity or goiter/nodularity (52 to 61% or 17 to 10%), TPOAb positiveness (76 to 57%, with mean levels halving from 1,707 to 813 U/ml). Similar or greater changes in Messina had occurred earlier and quicker, *viz.* between 1996 and 1998. Indeed, the number of HT patients doubled (97 to 225), the rate of subclinical or overt hypothyroidism within hypothyroid patients increased (55% in 1996 to 87% in 1997) or decreased (45 to 13%), the rate of variants nongoitrous/nonnodular or goitrous/nodular increased (21% in 1996 to 29% in 1997) or decreased (45 to 34%), the rate of TPOAb positiveness decreased (91% in 1996 to 81% in 1998, with mean levels halving from 1,373 in 1997 to 771 in 1998). Thus, thyroid autoimmunity is responding to environmental changes far more quickly than thyroid oncogenesis.

The direction of the difference noticed for HT (Messina>Catania) is reversed, but on a smaller scale, for TC (Catania>Messina) [11]. In a study that evaluated the epidemiology of TC during 2002–04 [11], the incidence of TC in Catania was 2.2-fold or 2.1-fold greater than in females or males from the other eight Sicilian provinces (1.95-fold or 1.52-fold *vs.* Messina). In particular, the rate of PTC was 94.1% in the Catania province and 86.2% in the rest of Sicily. Data did not change in 2005–06 (G. Pellegriti, personal communication). Should the other Sicilian provinces have an incidence of HT similar to that of Messina (hence, greater than Catania) over comparable years, then the intriguing scenario would be one of environmental changes that have increased the occurrence of both HT (concurrently modifying its presentation) and TC (without modifying its presentation). However, in certain areas (Catania) it was the neoplasia to be favored, while in other areas (rest of Sicily) it was the autoimmune disease to be favored. Perhaps, Messina and Catania shared a number of HT-associated environmental factors (or Messina was exposed to an higher dose of the same factors, and possibly earlier), but only Catania features TC-associated environmental factors (or shares them with the rest of Sicily, though at an higher concentration). The nature of environmental factors is beyond the scope of our work.

Two Turkish academic Divisions of Endocrinology [3], [13] studied the association between HT and TC. The study in Ankara compared FNAC diagnoses in 191 thyroid nodules of 164 HT patients with those in 713 nodules of 541 age-matched nonHT subjects; either group was observed in the years 2006–09 [3]. The study in Izmir, ≈500 Km away from Ankara, was conducted on 769 HT patients [13]. The Ankara cohort [3] is Messina-like while the Izmir cohort [13] is Catania-like. Indeed, compared to Izmir, the Ankara cohort had lower both F∶M ratio (8.6∶1 *vs.* 16.4∶1) and rate of positiveness for serum thyroid antibodies (TgAb = 69.5%, TPOAb = 80.5%. *vs.* >90% for either Ab). In the Ankara cohort [3], the rate of malignancy at FNAC (confirmed at histology) was 1.0% (HT patients) and 2.7% (nonHT patients). Though not significant (P = 0.147 by Fisher's exact test), the magnitude of the difference is almost 3.0-fold. By contrast, in the Catania-like Izmir cohort [13] the rate of malignancy (always PTC, as confirmed by histology) was 1.93%. This 1.93-fold greater difference between Izmir and Ankara is strikingly reminiscent of the difference between Catania and Messina (1.95-fold greater in women, and 1.52-fold greater in men) (11).

**Figure 6:**
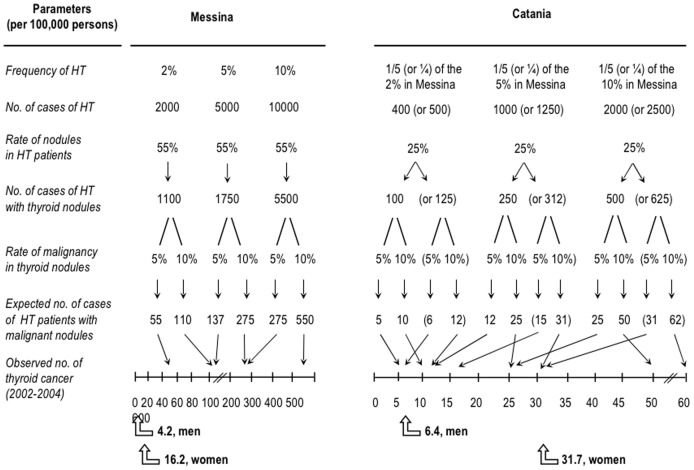
Expected epidemiology of thyroid cancer in patients with the nodular variant of Hashimoto's thyroiditis (HT) from the provinces of Messina and Catania (based on data reported here for the period 1995–2005) and observed epidemiology (years 2002–2004, as reported in ref. 11). The possible scenarios assume three rates of prevalence of HT in the general population (2, 5 or 10%) and two rates of malignancy for thyroid nodules (5 or 10%). The one-fourth or one-fifth lower magnitude of the HT prevalence in the Catania province is because the 1 to 4.5 ratio observed between Catania and Messina (742 and 3,409 cases, respectively) can be rounded off to either 1.0 to 4.0 or 1.0 to 5.0. The rates of thyroid nodules in HT patients (55% for Messina or 25% for Catania) were taken from the variant “thyroid nodules regardless of goiter” in Table 1 of the present paper. Expected numbers do not change substantially by referring to the period 2002–2004, because cases of HT were 352 in the Catania cohort and 1,351 in the Messina cohort (ratio of 1 to 3.84), with a rate of 26.4% and 56.5% for the variant “nodules regardless of goiter”.

If HT was a risk factor for TC, then the incidence of TC would have been greater in the province of Messina compared to Catania. And even more so, based on the said 10-fold greater number of HT patients with the variant “thyroid nodularity, regardless of goiter” in Messina, this being the appropriate comparison considering that cancer necessarily arise from thyroid nodules. The simulations in the Figure 6 suggest that this variant may well contribute to the overall number of TC observed in the Catania province whereas, in sharp contrast, the expected number of TC in Messina exceeds largely the observed number. Hence, one might conclude that, at least in given areas (*i.e.*, Messina), environmental conditions that trigger HT are protecting from TC. However, there could be another explanation. The alternative explanation takes into account that (i) the vast majority of TC is due to PTC, (ii) PTC is so relatively indolent that it may be discovered at autopsy, (iii) when PTC is clinically evident and associated with HT, it has a milder course. Thus, the number of real cases of PTC in Messina is greater than that observed, but it does not show up simply because PTC nodules might be so microscopic and devoid of suspicious characteristics that would escape attention (*viz*., investigation by FNAC) and/or would appear very late in life. Only a TC screening based on ultrasound-guided FNAC of all thyroid nodules in all HT patients with nodules and/or on autopsy can tell if the alternative interpretation is tenable. A final implication of our data is that studies across the world on the link between HT and TC [2]–[5] may yield contrasting results because geographical areas are heterogeneous, in that differing for the local epidemiology of HT, TC or both, further to differing for environmental conditions.

Our data on the goitrous/nonnodular variant are of interest because this index prevailed in Catania. Thus, exogenous factors exerted a diffuse growth stimulus for the thyroid parenchyma of HT patients in Catania, but a more focal stimulus in Messina so as to favor the clonal growth of thyrocytes that eventually formed thyroid nodules at a rate greater than in Catania. The thyroid of the Catania HT patients seems to be more resistant to such growth stimuli because the rate of the nongoitrous/nonnodular variant was greater than in Messina. This last difference is impressive considering that the Catania HT cohort had a greater prevalence of overt hypothyroidism, meaning a greater proportion of HT patients whose thyroid was exposed to higher serum levels of TSH.

One inference from having shown that data from two neighboring areas are not the photocopy of each other is that HT data from one area in a given period of time cannot be extrapolated to represent larger areas and wider periods. Study of the simultaneous changes of both thyroid autoimmunity and thyroid neoplasms in given geographical areas, but coupled with environmental studies, may prove helpful to identify the external factors that trigger either thyroid disorder.
